# The Indigenous Probiotic *Lactococcus lactis* PH3-05 Enhances the Growth, Digestive Physiology, and Gut Microbiota of the Tropical Gar (*Atractosteus tropicus*) Larvae

**DOI:** 10.3390/ani14182663

**Published:** 2024-09-13

**Authors:** Graciela María Pérez-Jiménez, Carina Shianya Alvarez-Villagomez, Marcel Martínez-Porchas, Estefanía Garibay-Valdez, César Antonio Sepúlveda-Quiroz, Otilio Méndez-Marín, Rafael Martínez-García, Ronald Jesús-Contreras, Carlos Alfonso Alvarez-González, Susana del Carmen De la Rosa-García

**Affiliations:** 1 Laboratorio de Fisiología en Recursos Acuáticos (LAFIRA), División Académica de Ciencias Biológicas, Universidad Juárez Autónoma de Tabasco, Carretera Villahermosa-Cárdenas Km. 0.5, Villahermosa 86039, Tabasco, Mexico; gjimenez9@outlook.com (G.M.P.-J.); carina.alvarez@ujat.mx (C.S.A.-V.); or cesar.sq@villahermosa.tecnm.mx (C.A.S.-Q.); otilio.mendez.marin@gmail.com (O.M.-M.); biologomartinez@hotmail.com (R.M.-G.); romeo_4500@hotmail.com (R.J.-C.); 2 Centro de Investigación en Alimentación y Desarrollo, A.C. Biología de Organismos Acuáticos, Hermosillo 83304, Sonora, Mexico; marcel@ciad.mx (M.M.-P.); estefania.garibay@ciad.mx (E.G.-V.); 3 Instituto Tecnológico de Villahermosa, Tecnológico Nacional de México, Carretera Villahermosa-Frontera, Km. 3.5, Ciudad Industrial, Villahermosa 86010, Tabasco, Mexico; 4 Laboratorio de Microbiología Aplicada (LABMIA), División Académica de Ciencias Biológicas, Universidad Juárez Autónoma de Tabasco, Carretera Villahermosa-Cárdenas Km. 0.5, Villahermosa 86039, Tabasco, Mexico

**Keywords:** probiotics, tropical gar, microbiome, physiology, gene expression, *Lactococcus lactis*

## Abstract

**Simple Summary:**

Microorganisms isolated from the intestinal microbiota of fish have demonstrated probiotic effects. Considering their beneficial impact on aquaculture, it is essential to develop feeding strategies that ensure the growth and health of aquaculture animals using isolated strains from fish. This study evaluated the effects of the administration of *Lactococcus lactis* PH3-05, a probiotic bacterium isolated from tropical gar adults (*Atractosteus tropicus*), on the growth, survival, digestion, intestinal morphology, gene expression, and intestinal microbiota of larvae of the same species. The results showed that supplementation with *L. lactis* PH3-05 significantly improved growth, survival, and digestive enzyme activity. In addition, this supplementation stimulated the expression of genes associated with the mucosal barrier and the anti-inflammatory response. Although no significant changes were observed in the overall composition of the intestinal microbiota, an increase in the abundance of *Lactobacillus* was recorded in the group treated with *L. lactis*. These findings suggest that *L. lactis* has potential as an indigenous probiotic to improve the health and growth of tropical gar larvae and could be implemented for native species of the region.

**Abstract:**

Probiotics in aquaculture hold promise for enhancing fish health and growth. Due to their increased specificity and affinity for their host, indigenous probiotics may offer isolated and potentially amplified benefits. This study investigated the effects of *Lactococcus lactis* PH3-05, previously isolated from adults of tropical gar (*Atractosteus tropicus*), on the growth, survival, digestive enzyme activity, intestinal morphology, expression of barrier and immune genes, and intestinal microbiota composition in the larvae of tropical gar. Larvae were fed with live *L. lactis* PH3-05 concentrations of 10^4^, 10^6^, and 10^8^ CFU/g for 15 days alongside a control diet without probiotics. Higher concentrations of *L. lactis* PH3-05 (10^6^ and 10^8^ CFU/g) positively influenced larval growth, increasing hepatocyte area and enterocyte height. The 10^6^ CFU/g dose significantly enhanced survival (46%) and digestive enzyme activity. Notably, the 10^8^ CFU/g dose stimulated increased expression of *muc-2* and *il-10* genes, suggesting enhanced mucosal barrier function and anti-inflammatory response. Although *L. lactis* PH3-05 did not significantly change the diversity, structure, or Phylum level composition of intestinal microbiota, which was constituted by Proteobacteria, Bacteroidota, Chloroflexi, and Firmicutes, an increase in *Lactobacillus* abundance was observed in fish fed with 10^6^ CFU/g, suggesting enhanced probiotic colonization. These results demonstrate that administering *L. lactis* PH3-05 at 10^6^ CFU/g promotes growth, survival, and digestive health in *A. tropicus* larvae, establishing it as a promising indigenous probiotic candidate for aquaculture applications.

## 1. Introduction

The growing demand for fish products has propelled aquaculture to the forefront of global food production to meet human needs [1]. In this context, ensuring the growth of fish during their culture, particularly in the technification processes, is crucial, which necessitates safeguarding the health of the organisms [2,3,4]. Therefore, the recent utilization of pre-and probiotics in balanced feeds as a more suitable alternative to boost the immune system and optimize the intestinal microbiome is significant. This approach has been reported to enhance digestive physiology, increase survival, and improve the health of organisms in culture [5,6,7,8]. The most used probiotic bacteria in aquaculture, many of them indigenous, belong to the genera *Bifidobacterium*, *Enterococcus*, *Bacillus*, *Lactobacillus*, *Lactococcus*, *Leuconostoc*, *Pediococcus* and *Weissella*, which have been isolated from the intestinal system of various fish species [9,10,11].

Among all the probiotic bacterial species, *Lactococcus lactis* stands out as one of the most promising. It is a lactic acid bacterium commonly found in the gastrointestinal tract of many fish species and possesses characteristics that make it a suitable probiotic. It can produce antimicrobial compounds, improve digestibility and nutrient absorption, and modulate immune responses, all contributing to improved fish health [12,13,14,15]. Additionally, this bacterium produces bacteriocins and enhances the biosynthesis of essential amino acids, further promoting fish health [16,17,18]. Given these characteristics, *L. lactis* holds great potential as an excellent probiotic candidate to promote the well-being of aquatic organisms.

Based on the above, several studies have shown that the inclusion of *L. lactis* has beneficial effects on survival, weight gain, and disease resistance by improving the immune system, increasing mucosal production, and enhancing intestinal microbial richness and diversity in different fish species. Notably, in the case of *Seriola dumerili* [19], the administration of *L. lactis* at a dose of 10^10^ CFU/g stimulated growth and reduced feed intake. In red seabream (*Pagrus major*), *L. lactis* (10^9^ CFU/g) promoted a significant increase in weight, weight gain (WG), specific growth rate (SGR), protein efficiency (PER) and survival, in addition to modulating the intestinal microbiota and strengthening the immune system [20]. Similar results have been obtained in common carp (*Cyprinus carpio*), where L. *lactis* (10^8^ CFU/g) improved immunity, resistance to pathogens, and productive parameters [21]. Likewise, in orange-spotted grouper (*Epinephelus codes)* and bastard halibut *(Paralichthys olivaceous*), supplementation with *L. lactis* has been shown to improve intestinal microbial diversity, intestinal barrier function, and growth [22,23].

In Southeastern Mexico, tropical gar (*Atractosteus tropicus*), a native ancestral fish species of economic and ecological importance, has been commercially cultivated for over 20 years [24]. As part of the development of balanced feeds and their optimization, the inclusion of various prebiotics (inulin, fructo-oligosaccharide, mannan-oligosaccharides, and β-glucans) has been evaluated, which have shown diverse beneficial effects in larvae and juveniles [25,26,27,28,29,30]. Additionally, in juvenile *A. tropicus*, only the probiotic yeast *Debaryomyces hansenii* has been evaluated, where no positive effects on growth and physiological variables were observed when included in high concentrations (10^14^, 10^15^, and 10^16^ CFU/Kg feed) in the balanced feed [31]. However, the use of probiotics during the larval stage in *A. tropicus* has not been evaluated. It is important to note that in *A. tropicus,* the larval stage shows variable survival rates, possibly due to several factors, such as the low adaptation to balanced food in the weaning process and the high cannibalism that occurs during this period [32,33,34]

On the other hand, a significant study is the one conducted by Méndez-Pérez [35], who characterized the intestinal microbial composition of juvenile and adult *A. tropicus*, where some bacterial species with probiotic potential were identified, such as *Cetobacterium*, *Aeromonas hydrophila* P5, *Aeromonas sobria* CP DC28, and *Lactococcus lactis*. This last bacterium was isolated and characterized as a suitable probiotic. Thus, this study evaluated the effects of adding *Lactococcus lactis* into weaning food on the growth, survival, digestive enzyme activity, gut morphology, and gene expression of *A. tropicus* larvae.

## 2. Materials and Methods

### 2.1. Indigenous Bacteria of A. tropicus

The Applied Microbiology Laboratory (DACBiol-UJAT) provided *Lactococcus lactis* PH3-05, which was isolated from the intestine of an adult male of *A. tropicus* (Gene Bank OK178269).

### 2.2. Obtaining the Bacterial Biomass

Biomass was obtained by a 72h culture in Brain Heart Infusion broth (BHI, Difco, Pittsburgh, PA, USA) with shaking at 140 rpm at 32 °C. The culture was centrifuged at 4000 rpm for 40 min, and the cell pellet was washed twice with sterile saline. The Colony-Forming Units (CFU) of the biomass obtained were quantified [36], and the number of CFU per *g* of wet biomass was reported. The antagonistic activity of *Lactococcus lactis* PH3-05 against pathogenic strains of fish was previously tested (Table S1).

### 2.3. Preparation of Experimental Diets

Four experimental diets were formulated following the protocol of [27]. Three diets were supplemented with L. lactis PH3-05 at 104, 106, and 108 CFU/g concentrations, and a control diet without probiotics (CD) (Table 1). The diets were prepared following the methodology of [37], and the bacteria were incorporated into the diets by dissolving the biomass in 200 mL of sterile water. The pelleted diets were dried at 40 °C for 15 h in a conventional oven and stored at 4 °C until use. The physical characteristics of the experimental diets were subjected to a grinding and sieving process to obtain an adequate particle size (500–800 µm) specifically for the morphological characteristics of the larvae’s mouth, as well as their nutritional requirements in terms of hardness and floatability.

All diets were analyzed for proximate analysis (humidity, ash, lipid, and protein) according to [38].

### 2.4. Viability of L. lactis PH3-05 in the Experimental Diets

At the beginning and end of the experiment, the CFU/g of bacteria in each experimental diet were quantified to confirm the concentrations and viability. A representative sample was obtained from each diet using the quartering method. One g of the diet was weighed and suspended in 9 mL of saline (0.85%), and exponential dilutions were made from 10^−1^ to 10^−10^. An amount of 100 µL of the different dilutions was inoculated in Agar (BHI) and added with an antifungal (natamycin) to reduce the fungi load in the formulated food. The inoculum was homogeneously dispersed with the help of a sterile Drigalsky loop; the plates were incubated for 24 h at 32 °C. Colonies were counted in dilution boxes with 10–30 colonies, and the CFU/g feed was calculated.

### 2.5. Experimental Design

This experimental design used 1200 *A. tropicus* larvae 5 days after hatching (DAH), with an average weight of 0.02 ± 0.00 g and total length of 1.81 ± 0.18 cm, obtained from the aquaculture farm “Otot Ibam”, Comalcalco, Tabasco. A total of 100 larvae per treatment were placed in 12 tanks of 70 L capacity, connected to a recirculation system driven by a 1HP water pump connected to a 1500 L reservoir with a biological filter and an automated sand filter and connected to an ultraviolet light lamp to minimize the possible bacterial discharge. Vat temperature (27.1 ± 0.1 °C), dissolved oxygen (5.5 ± 0.2 mg/L), and pH (7.3 ± 0.5) were monitored daily using an oximeter (YSI 85, Yellow Springs, OH, USA) and a potentiometer (HANNA HI 991001, Woonsocket, RI, USA).

Feeding was carried out with co-feeding during the first 5 DAH, providing *Artemia* sp. nauplii and microparticles of the experimental diets, including the control diet (approx. 350 μm); subsequently, the nauplius supply was eliminated, and only the balanced diets were administered until the end of the bioassay (20 DAH), adjusting the size of the microparticles according to the larvae growth (500–800 µm). Larvae were fed six times daily (00:00, 3:00, 7:00, 11:00, 15:00, and 19:00) until apparent satiation. Each tube was cleaned with partial water replacement daily (30%) after each feeding using the siphoning method. All treatments were evaluated in triplicate.

### 2.6. Evaluation of Growth and Survival Rate

At the beginning and end of the experiment, each larva was evaluated to determine the wet weight (g) with an analytical balance (Ohaus HH120, precision 120 ± 0.01 g, Shenzhen, China) and total length using photographs analyzed in ImageJ 1.5 software. At the end of the bioassay, the following productive parameters were determined: specific growth rate (SGR), [(ln final weight − ln initial weight)/days] × 100; weight gain (WG), final weight (g) − initial weight (g); and survival rate (S), (number of final fish/number of initial fish) × 100.

### 2.7. Sample Collection

At the end of the bioassay, the larvae were sacrificed by thermal shock. The head and tail were removed, and the larvae were washed with water. To ensure representativeness, the following samples were obtained per replicate. Three larvae were kept at −80 °C to determine enzyme activity. Three larvae were preserved in RNAse-free tubes for metagenomic analysis, three for gene expression analysis (RNAlater solution, Ambion, Norristown, PA, USA), and three for histological analysis, which were fixed in Davison solution. The study was carried out under the Helsinki Declaration and the protocol authorized by the Ministry of Agriculture, Livestock, Rural Development, Fisheries and Food (SAGARPA), Mexico, NOM-062-Z00-1999 [39].

### 2.8. Enzyme Activities Quantification

The multienzyme extract was obtained from the pool of three larvae per replicate in 50 mM L^−1^ Tris-HCl, pH 7.5, using a tissue homogenizer (Ultra Turrex IKA T18 Basic, Staufen, Germany) and centrifuged at 14,000 rpm for 15 min. The supernatant was stored at −80 °C. Soluble protein was quantified using 50 µL Bradford as a substrate and quantified at 592 nm according to the Bradford technique [40]. Acid protease activity was quantified using 1% hemoglobin as a substrate in 0.1 M Glycine HCl buffer, pH 2. The absorbance was measured on a microplate reader (xMark, Bio-Rad, Hercules, CA, USA) at 280 nm, according to [41]. The technique of [42] determined alkaline protea with 1% casein as a substrate and 100 mM Tris-HCl and CaCl_2_ 10 mM at pH 9, and absorbance was measured at 280 nm. Trypsin activity was quantified with 1 mM BAPNA as a substrate (Nα-Benzoyl-DL-Arginine-P-nitroanilide) in 50 mM Tris-HCl and 10 mM CaCl_2_ at pH 8.2, and absorbance was measured at 410 nm, according to the technique of [43]. Following the method of [44], chymotrypsin activity was determined, SAPNA 1.25 mM was used as a substrate (135 µL) at pH 8.2, and absorbance was measured at 410 nm. Lipase activity was performed with the methodology of [45] using 4-nitrophenyl palmitate as a substrate, pH 7.4, and 6 mM sodium taurocholate, and absorbance was measured at 540 nm. To quantify leucine aminopeptidase activity, 0.1 M leucine p-nitroanilide at pH 7.2 was used as a substrate and quantified at 405 nm absorbance, following the method of [46]. The activities of all enzymes were calculated with the following equations: (1) units per mL (U mL^−1^) = (Δabs × final volume of the reaction (mL)) × (MEC × time (min) × enzymatic extract volume (mL)^−1^); (2) units per mg of protein (U mg protein^−1^) = units per mL × mg of soluble protein^−1^, where MEC is the molecular extinction coefficient.

### 2.9. Histological Analysis

The samples were dehydrated in different concentrations of ethyl alcohol (50, 70, 80, 96, and 100%, OH 100%-Xylol). They were then embedded in paraffin. Cross sections of 7 µm thickness were obtained with a sliding microtome (Leica, Reichert-Jung, Hn40, Deer Park, IL, USA) and stained with hematoxylin and eosin (H-E). The slices were examined under a Zeiss optical microscope (Axio-star Plus, Oberkochen, Germany), the photographs were taken with a digital camera (Zeiss, Axiocam MRc 5, Oberkochen, Germany), and the morphometric measurements were taken with Zen 2.3 software. At the intestinal level, the height of the enterocytes (µm) and the number of fatty hepatocytes per area were quantified (50 and 30 measurements per individual, respectively).

### 2.10. RNA Extraction, Reverse Transcription, and Gene Expression Analysis

Total RNA was extracted using the Trizol technique (Invitrogen, Waltham, MA, USA) according to the manufacturer’s instructions. The concentration and purity of a single RNA were determined in a spectrophotometer (A260/280) (Jenway GenovaNano, Cole-Parmer, Staffordshire, UK). One microgram of RNA was reverse transcribed into cDNA in a thermal cycler (Mastercycle nexus GSX1, Eppendorf, Hamburg, Germany) using the High-Capacity cDNA Reverse Transcription Kit (Maxima First Strand cDNA Synthesis Kit for RT-qPCR, Thermo Scientific, Waltham, MA, USA) in a final volume of 20 µL, following the manufacturer’s recommendations. The expression of two genes associated with intestinal barrier integrity, *muc-2* (intestinal mucus layer protein) and *zo-2* (tight junction protein), and two immune system genes, *il-8* (proinflammatory cytokine) and *il-10* (anti-inflammatory cytokine), were evaluated (Table 2). The qPCR reactions were carried out using 5 µL of Eva Green supermix (BioRad, Hercules, CA, USA), 4.5 µL of cDNA, and 0.5 µL of primers mix (3 mM) at a final volume of 10 µL per reaction. The β-actin gene was used as a reference gene [47]. The qPCR was performed on a CFX96TM real-time thermocycler (BioRad, Hercules, CA, USA) using the following conditions: a 10 min denaturation cycle at 95 °C followed by 40 cycles of 15 s at 95 °C and 1 min at 60 °C. Relative gene expression changes were calculated using the 2^−ΔΔCT^ method [48].

### 2.11. DNA Isolation from Gut Microbiota and Preparation of 16S rRNA Gene Libraries and Sequencing

Genomic DNA extractions were performed using a commercial kit (DNeasy PowerLyzer PowerSoil Kit, QIAGEN, Hilden, Germany). DNA concentration was quantified using the Qubit 3.0 fluorometer and the ds-DNA BR Assay kit (Invitrogen from Thermo Fisher Scientific). DNA integrity was verified by 1% agarose gel electrophoresis.

The V3 hypervariable region of the bacterial 16S rRNA gene was PCR amplified for library preparation using gene-specific primers V3-338f and 533r [49]. PCR amplification and second PCR were performed using the Nextera XT index (Illumina, San Diego, CA, USA). All PCR products were followed by an optimized clean-up step using Agencourt AMPure XP beads according to the protocol published by Illumina. Finally, V3 libraries were sequenced with a MiSeq v3 reagent kit (300 cycles) using the MiniSeq platform (Illumina, San Diego, CA, USA), and 2 × 150 cycles of paired-end sequencing were performed.

### 2.12. Data Analysis and Statistics

First, normality (Kolmogorov–Smirnov) and homoscedasticity (Bartlett) tests were performed on the growth data, digestive enzyme activities, and histological measurements, all meeting the required assumptions. Consequently, a one-way ANOVA followed by Tukey’s post hoc test was performed. The Kruskal–Wallis test and Nemenyi’s post hoc test were used for gene expression analyses to identify significant differences. All data were statistically analyzed using GraphPad Prism 8 software (GraphPad Software, San Diego, CA, USA) with a significance value of 0.05.

## 3. Results

### 3.1. Growth Indexes and Survival Rates

At the end of the bioassay (20 DAH), larvae fed with *L. lactis* PH3-05 10^8^ CFU/g obtained the highest final weight (0.041± 0.00 g), followed by larvae with 10^6^ CFU/g (0.040 ± 0.00 g) of *L. lactis* PH3-05. Both treatments showed significant differences (*p* < 0.05) with larvae fed with *L. lactis* PH3-05 10^4^ CFU/g and CD (Table 3). The most significant total length was shown in larvae fed with all three *L. lactis* PH3-05 supplementations (*p* < 0.05); however, the 10^8^ CFU/g treatment showed the greatest total length (2.38 ± 0.09 cm). SGR and WG were highest in larvae fed with the diets supplemented with 10^8^ CFU/g of *L. lactis* PH3-05 (2.83 ± 0.003 and 52.14 ± 0.06, respectively), followed by those fed with 10^6^ CFU/g (2.75 ± 0.15 and 50.33 ± 3.50, respectively) of *L. lactis*, showing significant differences (*p* < 0.05) with *L. lactis* 10^4^ CFU/g and CD larvae. The highest survival rate was presented in the larvae of treatment 10^6^ CFU/g of *L. lactis* PH3-05 with 46.36 ± 4.34%, showing significant differences (*p* < 0.05) with the rest of the treatments (Table 3).

### 3.2. Digestive Enzyme Activity

The specific activity of acid and alkaline protease was significantly higher in larvae fed with 10^6^ CFU/g of *L. lactis* PH3-05 compared to the rest of the treatments (*p* < 0.05). Regarding CD, trypsin activity was higher (*p* < 0.05) in larvae fed 10^8^ CFU/g of *L. lactis* PH3-05. On the other hand, larvae fed 10^4^ and 10^6^ CFU/g of *L. lactis* PH3-05 showed higher chymotrypsin activity than the rest of the treatments. Likewise, administering 10^6^ CFU/g of *L. lactis* PH3-05 favored significantly (*p* < 0.05) higher lipase activity than the rest of the treatments. Finally, leucine aminopeptidase activity showed a significant increase for larvae fed with 10^4^ CFU/g of *L. lactis* PH3-05 (*p* < 0.05) for those fed with CD (Table 4).

### 3.3. Histological Analysis

**Figure 1:**
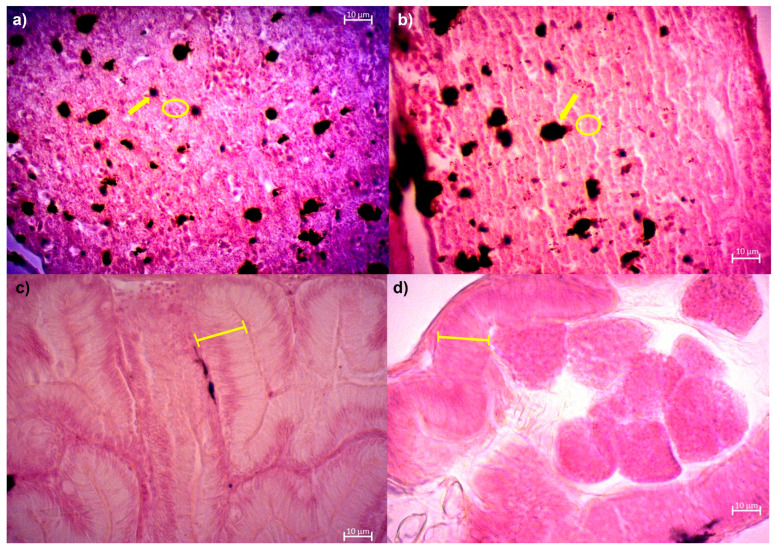
Representative images of the liver and digestive system of *A. tropicus* larvae treated with CD and 10^6^ CFU/g of *L. lactis* PH3-05: (**a**) CD, (**b**) 10^6^ CFU/g of *L. lactis* PH3-05. The liver images show melanomacrophagic centers (yellow arrow) and hepatocytes (circle). Images of the intestine display the height of enterocytes (yellow line) of *A. tropicus* larvae: (**c**) CD, (**d**) 10^6^ CFU/g of *L. lactis* PH3-05.

The livers of the larvae fed with 10^6^ and 10^8^ CFU/g of *L. lactis* showed a significantly higher percentage of melanomacrophagic centers (MMC) compared to those fed with the CD (*p* < 0.05) (Table 5). In the intestine, the height of enterocytes was significantly higher in larvae fed with 10^6^ CFU/g of *L. lactis* (*p* < 0.05) compared to all other treatments, especially those fed with CD, which exhibited the lowest enterocyte height (Table 5, Figure 1a,b).

### 3.4. Gene Expression

**Figure 2:**
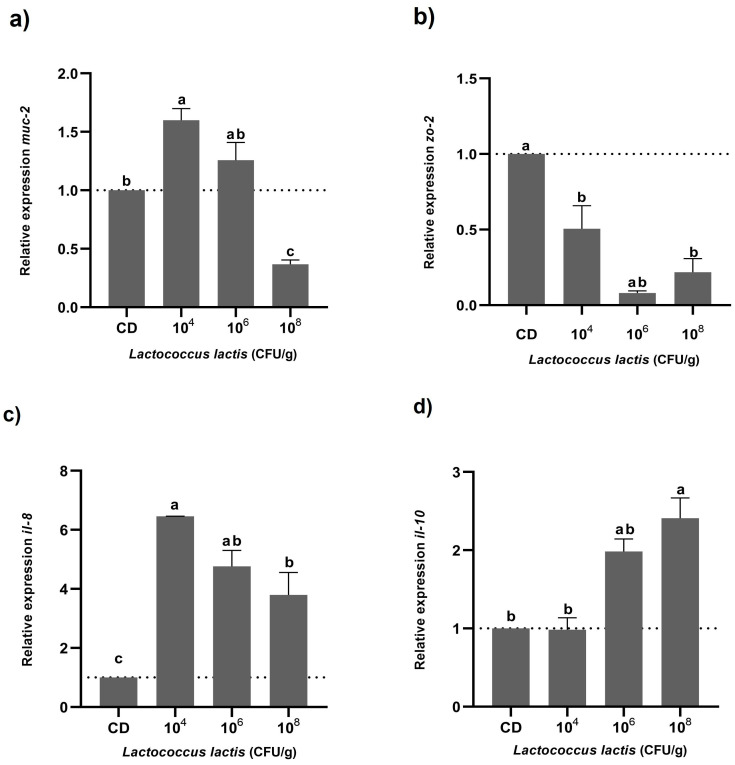
Relative expression levels of intestinal barrier function and immune system genes in *A. tropicus* larvae fed with *L. lactis* PH3-05 supplementation (10^4^, 10^6,^ and 10^8^ CFU/g) and the control diet. Values are mean ± SD. Data are presented as fold-change relative to control diet samples (set to 1). Significant differences between treatments are indicated by letters (*p* < 0.05). (**a**) Mucus layer protein (*muc-2)*; (**b**) Tight junction protein (*zo-2*); (**c**) Pro-inflammatory cytokine (*il-*8); (**d**) Anti-inflammatory cytokine (*il-*10).

The relative expression of *muc-2* significantly increased (*p* < 0.05) in larvae fed with 10^4^ and 10^6^ CFU/g of *L. lactis* compared to the other treatments (Figure 2a). In contrast, *zo-2* expression was significantly down-regulated (*p* < 0.05) in larvae across all three *L. lactis* supplementation groups (Figure 2b). Additionally, all three *L. lactis* supplementations induced a significant increase in *il-8* expression (*p* < 0.05) compared to those fed with CD (Figure 2c). However, the relative expression of *il-10* also showed a significant increase in larvae fed with 10^6^ and 10^8^ CFU/g of *L. lactis* (*p* < 0.05) compared to those fed with the other treatments (Figure 2d).

### 3.5. Classification and Taxonomy of the Bacterial Taxonomic Profile

The metagenomic analysis was conducted using 16S rRNA gene sequencing, yielding 765,724 sequences. After removing chimeras, 26,679 sequences were classified into 461 OTU (operational taxonomic units).

**Figure 3:**
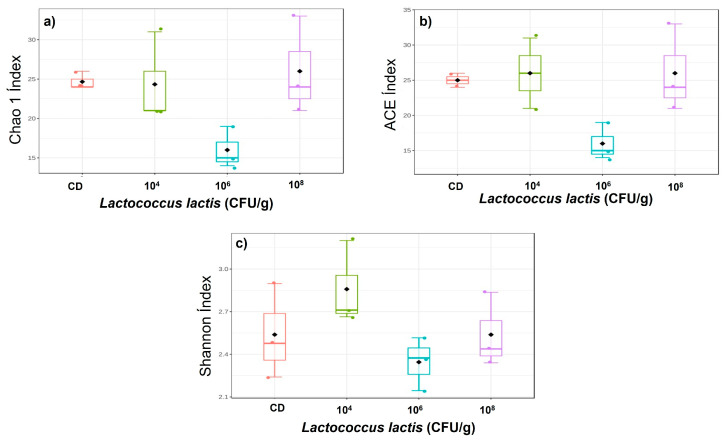
Alpha diversity of gut microbiota in *A. tropicus* larvae treated with *L. lactis* PH3-05 supplementations (10^4^, 10^6,^ and 10^8^ CFU/g) and control diet: (**a**) Chao 1, (**b**) ACE, and (**c**) Shannon–Weaver indexes were calculated from the ASVs.

The alpha diversity indices are presented in Figure 3. According to Chao 1 (*p* < 0.0897), ACE (*p* < 0.1331), and Shannon–Weaver (*p* < 0.2260) estimators, there were no significant differences in alpha diversity larvae fed with the three *L. lactis* supplementations and the CD.

**Figure 4:**
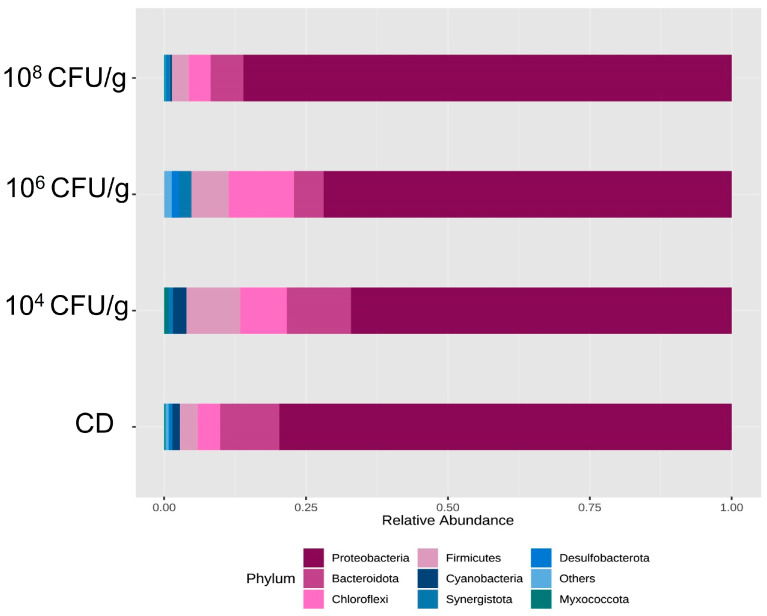
A relative abundance of bacterial phyla is present in the intestinal microbiota of *A. tropicus* larvae fed with *L. lactis* PH3-05 (10^4^, 10^6,^ and 10^8^ CFU/g) and a control diet.

Figure 4 shows the composition of the intestinal microbiota of *A. tropicus* larvae, which consists of the phyla Proteobacteria, Bacteroidota, Firmicutes, Chloroflexi, and Desulfobacterota. Proteobacteria was the most dominant Phylum in all treatments. In this aspect, larvae fed 10^8^ CFU/g of *L. lactis* PH3-05 showed the highest relative abundance of Proteobacteria with 78.92%, followed by those fed with CD (71.35%). In comparison, in larvae fed with 10^6^ CFU/g of *L. lactis* PH3-05, the abundance was 58.20%, and finally, larvae fed with 10^4^ CFU/g of *L. lactis* PH3-05 showed the lowest abundance of Proteobacteria with 49.85%. 

The highest relative abundance of Bacteroidota was found in larvae fed with CD, with 13.53%, followed by larvae fed with 10^4^ CFU/g of *L. lactis*, with 13.31%. Finally, the lowest relative abundances were presented in larvae fed with 10^6^ CFU/g of *L. lactis* (8.14%) and 10^8^ CFU/g of *L. lactis* (7.93%), respectively. The relative abundance of the phylum Firmicutes was highest in larvae fed 10^4^ CFU/g of *L. lactis* (12.66%) and 10^6^ CFU/g of *L. lactis* PH3-05 (7.58%). In contrast, larvae fed the CD and 10^8^ CFU/g of *L. lactis* PH3-05 showed the lowest relative abundances (5.54% and 4.755%), respectively. The highest abundance of phylum Chloroflexi occurred in larvae fed with 10^6^ CFU/g of *L. lactis* PH3-05 (14.62%) and 10^4^ CFU/g of *L. lactis* (9.36%). Again, the lowest relative abundances were obtained with larvae fed with CD and 10^8^ CFU/g of *L. lactis* (3.82% and 3.54%), respectively. 

At the order level, Burkholderiales, Cytophagales, Bacteroidales, Sphinogomonadales, Pseudomonadales, and Lactobacilliales were the most representative in all treatments. The most representative families in all treatments were Oxalobacteraceae, Comamonadaceae, Sphinogomonadaceae, Anaerolineaceae, Pseudomonadaceae, Lactobacilliaceae, and Caulobacteraceae.

**Figure 5:**
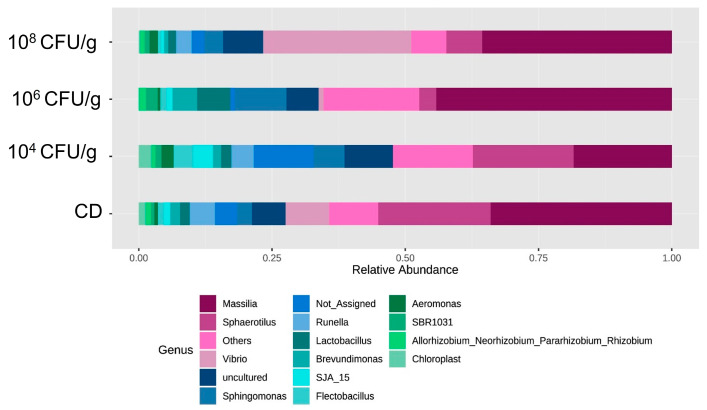
Relative abundance of the bacterial genus is present in the intestinal microbiota of *A. tropicus* larvae fed with *L. lactis* (10^4^, 10^6^, and 10^8^ CFU/g) and a control diet.

Figure 5 shows the relative abundance at the genus level of the intestinal microbiota of *A. tropicus* larvae, where the most representative genera are *Masillia*, *Sphaerotilus*, *Vibrio*, *Sphingomonas*, *Runella*, and, to a lesser extent, *Pseudomonas*, *Lactobacillus*, and *Brevundimonas*. *Massillia* has the highest relative abundance in larvae fed with 10^6^ CFU/g of *L. lactis*, PH3-05 31.03%, followed by those fed 10^8^ CFU/g of *L. lactis* PH3-05 (26.70%). In larvae fed with CD, 21.23% of relative abundance was obtained, and the lowest proportion was for larvae fed with 10^4^ CFU/g of *L. lactis* PH3-05 (12.94%). The genus *Vibrio* was present only in larvae fed 10^8^ CFU/g of *L. lactis* (18.36%) and those fed with CD (8.29%), respectively. The genus *Runella* presented 4.35% relative abundance in larvae fed with the CD and in smaller proportions in larvae fed with 10^4^ CFU/g and 10^8^ CFU/g of *L. lactis* (2.12% and 2.22%, respectively), while in larvae fed with 10^6^ CFU/g of *L. lactis* PH3-05, the presence of this genus was not detected.

The highest relative abundance of the genus *Lactobacillus* was shown in larvae fed 10^6^ CFU/g of *L. lactis* PH3-05 (4.29%), and the lowest relative abundance was observed in larvae fed 10^8^ CFU/g of *L. lactis* PH3-05 (1.09%). Bacteria of the genus *Pseudomonas* showed a relative abundance of 6.38% in larvae fed with 10^6^ CFU/g of *L. lactis* PH3-05 and the lowest abundance in larvae fed with 10^8^ CFU/g of *L. lactis* PH3-05 (1.23%). The genus *Sphingomonas* recorded the highest relative abundance of the larvae fed with 10^4^ CFU/g (3.82%), 10^6^ CFU/g (6.33%), and 10^8^ CFU/g (4.60%) of *L. lactis*, while the lowest relative abundance was detected in larvae fed with CD with 2.18%. Bacteria of the genus *Brevundimonas* presented the highest abundance in larvae fed with 10^6^ CFU/g of *L. lactis* PH3-05 (3.44%), which decreased in larvae fed with 10^4^ CFU/g (1.42%), CD (1.17%), and 10^8^ CFU/g of *L. lactis* PH3-05 (1.02%).

### 3.6. Bacterial Community Structure through Beta Diversity

Our study, based on taxonomic assignment and a multivariate analysis of variance (PERMANOVA) with 4999 permutations, revealed a crucial finding: there were no significant differences in beta diversity indexes between the treatments with *L. lactis* PH3-05 and the CD.

**Figure 6:**
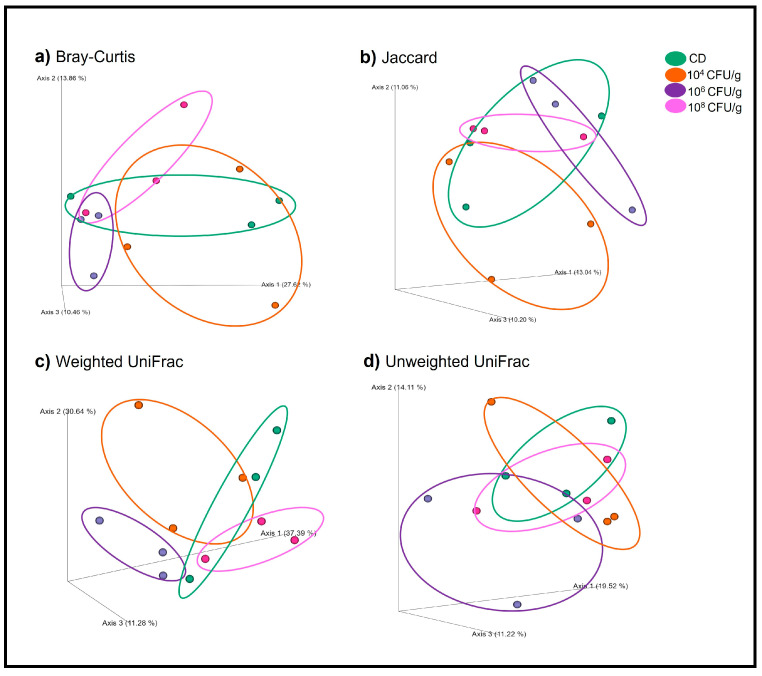
Principal coordinate analysis (PCoA) based on beta diversity analyses with Bray–Curtis (**a**), Jaccard (**b**), Weighted Unifrac (**c**), and Unweighted Unifrac (**d**) indexes of gut bacterial profiles of *A. tropicus* larvae treated fed *L. lactis* PH3-05 (10^4^, 10^6^, and 10^8^ CFU/g) and a control diet.

Figure 6 illustrates the precision of our research methods through principal coordinate analyses (PCoA) with beta diversity indices. The Bray–Curtis similarity (Figure 6a) showed 51.94% clustering (*p* < 0.105), while the cumulative variance by JACCARD (Figure 6b) showed a value of 34.3% distance between treatments (*p* < 0.12). The metrics of phylogenetic distances through UNIFRAC presented values of 79.31% and 44.86% of the total variance in the weighted (WEIGHTED *p* < 0.0212) (Figure 6c) and unweighted (UNWEITHED *p* < 0.6596) (Figure 6d) analyses, respectively. These measurements confirm that none of the indices showed significant differences between the treatments with *L. lactis* and the CD.

### 3.7. Predicted Metabolic Functions of the Intestinal Microbiota from KEGG

**Figure 7:**
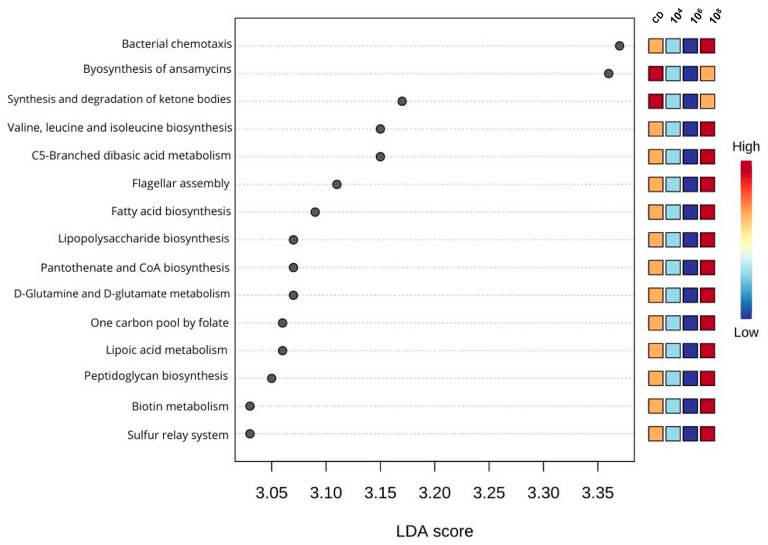
Heat map of microbial functions in the digestive tract of *A. tropicus* larvae fed with *L. lactis* PH3-05 (10^4^, 10^6^, and 10^8^ CFU/g) and a control diet. Predictions are based on level 3 functional annotations using the KEGG database.

An enhanced microbial function was detected in larvae fed with 10^8^ CFU/g of *L. lactis* compared to those fed with the CD, as determined by Lefse analysis. Enrichment functions included bacterial chemotaxis, the biosynthesis of valine, leucine, and isoleucine, branched dibasic acid-C5 metabolism, flagellar assembly, fatty acid and lipopolysaccharide biosynthesis, pantothenate, and CoA synthesis. Additionally, functions related to D-glutamine and D-glutamate metabolism, biotin metabolism, lipid metabolism, carbon deposition via folate, peptidoglycan biosynthesis, and the sulfur relay system were also enriched. Conversely, larvae fed with the CD exhibited higher microbial functions related to ansamycin synthesis and the degradation of ketone bodies (Figure 7).

## 4. Discussion

### 4.1. Growth Indexes and Survival Rate

Probiotics have successfully impacted aquaculture and have been considered a functional feed additive for cultured organisms [50].

Thus, the administration of *L. lactis* PH3-05 has provided positive results in different fish species, such as Nile tilapia (*Oreochromis niloticus*), where higher WG and higher survival were obtained [51]. In bastard halibut (*Paralichthys olivaceus*), including a concentration of 10^8^ CFU/mL favored higher weight gain, feed efficiency, SGR, PER, and condition factor [52]. Likewise, the administration of 10^8^ CFU/g of *L. lactis* L19 improved the growth, WG, feed efficiency index, SGR, and PER in snakehead fish (*Channa argus*) [53]. Similarly, a 10^8^ CFU/g concentration of *L. lactis* HNL12 in humpback grouper (*Cromileptes altivelis*) favored growth, total length, percentage weight gained, and SGR [54].

The proper administration of *L. lactis* improves the digestibility and absorption of nutrients provided in the diet, increasing productivity values and survival rates in several fish species [55,56]. In addition, it improves digestive enzymatic activities and promotes more significant growth and development of fish [57].

Because they produce short-chain fatty acids, which enterocytes use as an energetic substrate to maintain intestinal integrity, homeostasis, and digestive function, this result is consistent with our results by significantly promoting final weight, total length, SGR, WG, and survival in fish treated with 10^6^ CFU/g.

### 4.2. Digestive Enzyme Activity

Certain probiotics, such as *L. lactis*, have been reported to play a crucial role in the digestion of macronutrients in balanced diets fed to fish, which has been linked to increased hydrolysis and improved nutrient absorption in some fish species [21,58,59]. The presence of *L. lactis* has also been reported to increase the digestive enzyme activity of fish, in addition to the complementary hydrolysis due to the action of exogenous bacterial enzymes, which favor the hydrolysis of macromolecules (proteins, lipids, and carbohydrates). Thus, the bacterial pre-hydrolysis of feed nutrients and increased digestive enzyme activity in fish improve their growth and enhance their metabolic functions [60,61]. Our study observed that administering 10^8^ CFU/g of *L. lactis* PH3-05 in *A. tropicus* larvae significantly decreased digestive enzyme activity (acid protease, alkaline protease, chymotrypsin, lipase, and leucine aminopeptidase) compared to CD-fed larvae.

In this regard, the addition of probiotics has been shown to significantly improve the regulation of amino acids, fatty acids, vitamin metabolism, and digestive enzyme synthesis in fish, offering a promising avenue for enhancing fish nutrition [62]. It has also been shown that the metabolic processes of probiotics can produce exogenous enzymes that benefit the host organism by supplementing digestive enzyme activity and pre-digesting the feed provided to the fish, thereby improving nutrient absorption. However, improved absorption will depend on the appropriate concentration of the probiotic [63]. For example, the use of a probiotic consortium (*Bacillus subtilis*, *Lactobacillus acidophilus*, *Clostridium butyricum*, and the yeast *Saccharomyces cerevisiae*) in *O. niloticus* led to an increase in trypsin-like and amylase activities [64].

Similarly, the application of a commercial probiotic (PrimaLac^®^: *L. acidophilus*, *Lactobacillus casei*, *Enterococcus faecium*, and *Bifidobacterium thermophilus*) resulted in a significant increase in amylase, protease, and alpha-glucosidase enzyme activities in Caspian white fish (*Rutilus frisii kutum*) [65]. In the case of *L. lactis*, when administered in rainbow trout (*Oncorhynchus mykiss*), trypsin, lipase, and alkaline digestive protease enzyme activities were highly detected [64]. The same is observed in *C. carpio*, where digestive enzyme activity increased (amylase, lipase, and protease), along with several production parameters, immune system enzymes, and antioxidant activity [66].

### 4.3. Histological Analysis

Our research found that the use of *L. lactis* PH3-05 significantly increases the height of the enterocytes in *A. tropicus* larvae, allowing for a larger nutrient absorption area and, consequently, higher growth. These results align with findings in *O. niloticus*, where the administration of *L. lactis* (10^7−8^ CFU/g diet) resulted in increased villi length and muscle layer thickness compared to a diet without probiotics [67]. Similarly, *L. lactis* administration (10^8^ CFU/g) in the same species increased the density and length of intestinal microvilli compared to the control treatment [54]. In gilthead sea bream (*Sparus aurata*), the administration of *L. lactis* (2 and 5 × 10^9^ CFU/kg) reduced intestinal inflammatory processes and improved microbial composition [68].

Additionally, the hepatocyte area in *A. tropicus* larvae increased with *L. lactis* (10^8^ CFU/g). These results are consistent with those reported by [69], who observed in vitro that *L. lactis* increases hepatocyte proliferation and promotes liver cell protection in snakehead (*Channa argus*). Similarly, in *O. niloticus*, an increase in lipid accumulation in hepatocytes was observed with commercial probiotics (C.A. growth^®^ and Tonolest^®^) [70]. Furthermore, our research detected an increase in the presence of MMC in the liver with *L. lactis* PH3-05 treatments. This finding coincides with observations in *O. niloticus* when a probiotic (*Bacillus* spp.) was administered, leading to increased MMC in the spleen. This response is related to the organism’s physiological reaction to the probiotic, which is perceived as a potential pathogen, thus activating the immune system to prevent possible infection [71].

Similarly, in *O. niloticus*, the number of MMC increased when a commercial symbiotic was supplied, and the fish were challenged with *Pseudomonas fluorescens* [72]. A similar increase in MMC was observed when juveniles of the same species were supplemented with the probiotic *Pseudomonas putida* and challenged with *Aeromonas hydrophila* [73]. Thus, the increase in MMC is associated with the presence of the probiotic and is maximized when fish are challenged with pathogenic bacteria. However, the *A. tropicus* larvae in our study were not challenged by any known pathogen. Therefore, further research is needed to explore the effect on immune capacity and its relationship with the increase in MMC in this species.

### 4.4. Gene Expression

Probiotics, known for their potential to modulate the immune system and gut microbiota and their promising antagonistic effect against pathogenic microorganisms [74], represent a significant area of interest in immune health research.

In *A. tropicus* larvae, the administration of *L. lactis* PH3-05 (10^4^ and 10^6^ CFU/g) resulted in a significant increase in the relative expression of *muc-2*, suggesting an enhancement of the protective intestinal barrier. More importantly, this increase also indicates an activation of the immune system, as the mucus layer is the first line of defense against the translocation of toxic or pathogenic organisms. This finding aligns with the observations of [75], who also noted a similar increase in *muc-2* expression in response to a different stimulus. The mucus layer, secreted by goblet cells in the epidermis of fish, contains protective elements, such as glycoproteins, lysozymes, and immunoglobulins, among other antimicrobial compounds [76,77]. It serves multiple functions, including resistance and protection against infections, ionic and osmotic regulation, excretion, and nutrition absorption. This boost in mucus production can be interpreted as an active reinforcement of the immune system, highlighting the role of the mucus layer as the primary physical defense against harmful pathogens in the intestinal barrier.

On the other hand, the underexpression of the *zo-2* gene in larvae fed with *L. lactis* PH3-05 is a significant finding. This under-expression may be promoting the synthesis of beneficial metabolites, such as bacteriocins, γ-aminobutyric acid, ornithine, exopolysaccharides, and mannitol, among others [78], thereby eliminating the need for larvae to express *zo-2*. As a result, larvae fed with *L. lactis* PH3-05 experienced better growth. This finding is particularly noteworthy as a decrease in the expression of the *zo-2* gene has been reported to reduce intestinal function and can cause certain intestinal disorders in fish [79], which did not occur in *A. tropicus*. These results significantly enhance our understanding of the regulatory mechanisms of tight junction proteins, which are crucial in strengthening the intestinal barrier in *A. tropicus* in its larval stage.

Furthermore, the administration of *L. lactis* has been shown to improve the immune system directly [61]. In that sense, cytokines, the messenger proteins responsible for emitting the first warning signals of the immune system in response to harmful events, play a crucial role in the host organism’s defense [80,81]. Different strains of *Lactobacillus* and *Bifidobacteria* are known to release or increase the expression of *il-8* or *il-10* during normal mucosal conditions or inflammatory processes, helping to neutralize or prevent harmful stimuli [82]. In our study, *A. tropicus* larvae fed with *L. lactis* PH3-05 supplementation showed a higher expression of *il-8*, suggesting adaptation to the environment and a formulated diet and microbial colonization. In addition, it has been reported that certain strains of lactic acid bacteria can prevent an inflammatory response by activating the CD14 glycoprotein of epithelial cells [82].

Furthermore, the anti-inflammatory cytokine il-10 expression, vital for maintaining mucosal immune homeostasis in the intestinal tract [83], was significantly enhanced by *L. lactis* PH3-05 in *A. tropicus* larvae. Our results show a substantial increase in the expression of *muc-2* (10^4^ and 10^6^ CFU/g) and *il-10* (10^6^ and 10^8^ CFU/g) in *A. tropicus* larvae fed *L. lactis*, which is consistent with the findings from crucian carp (*Carassius carassius*) fed with *L. lactis* PH3-05 supplements. These carp showed a significant immune system response after exposure to *Aeromonas hydrophila*, marked by increased expression of anti-inflammatory interleukin (*il-11*) and the gene related to the reinforcement of the intestinal barrier (*zo-1*). The overexpression of INF-γ, IL-1β, and TNF-α further indicates that *L. lactis* administration effectively reduces intestinal inflammation caused by exposure to pathogen bacteria [84]. The high resistance to pathogenic bacteria is attributed to the probiotic ability of *L. lactis* PH3-05 to modulate the immune system by releasing certain antimicrobial compounds. Also, *il-11* expression suggests that the organism can protect and restore the gastrointestinal mucosa [85]. In Nile tilapia (*Oreochromis niloticus*), the inclusion of *L. lactis* strengthens the immune system, leading to increased expression of immune-related genes, specifically tumor necrosis factor (TNF-α) and interferon-gamma (IFN-γ), after exposure to the pathogen *Streptococcus agalactiae* [54]. This promising result opens potential applications of *L. lactis* PH3-05 in enhancing immune responses. However, the interaction between the gut microbiota and the immune system also involves specific metabolites secreted by the microbiota, which are absorbed by enterocytes and transported to the bloodstream and systemic lymphoid tissues. These metabolites can regulate host immune responses, play roles in inflammatory signaling, and interact directly or indirectly with host immune cells [86].

### 4.5. Gut Microbiome

The gut microbiome’s microorganisms play essential roles in various metabolic, physiological, and immunological functions with the host organism [14].

Administering *L. lactis* PH3-05 (10^6^ CFU/g) to *A. tropicus* larvae promotes an increase in the abundance of *Lactobacillus* in the intestinal microbiota. Previous studies [35] characterized the gut microbial composition of *A. tropicus* in both female and male juveniles and adults. The overall results showed that Fusobacteria is the most dominant phylum (42.26%), followed by Proteobacteria (31.40%), Firmicutes (12.96%), and Bacteroides (11.79%). Therefore, it can be considered a central gut microbial composition in *A. tropicus* at these stages. However, our results showed that the larval stage of *A. tropicus* is mainly composed of Proteobacteria and Bacteroides. The presence and function of Proteobacteria have been reported to increase the expression of genes related to RNA processing, degradation, the outer membrane, and lipopolysaccharide synthesis [87], which play roles in degrading Gram-negative (often pathogenic) microorganisms and enhancing the immune system. Bacteria belonging to the phyla Bacteroidetes and Proteobacteria could enhance metabolic and immune function and induce immune responses in the host, suggesting a possible relationship between these phyla and fish growth and immunity [88]. Additionally, the abundance of Proteobacteria may contribute to digestive functions in healthy fish [6].

This finding underscores the importance of our study in understanding the bacterial community structure. In this context, our research on *A. tropicus* larvae has revealed intriguing findings that open new avenues for further exploration. The Proteobacteria genus, particularly *Massillia* and *Sphaerotilus*, showed the highest abundance, while potentially harmful genera like *Vibrio* and *Aeromonas* decreased. This finding suggests potential future studies to further explore these genera’s role in the larvae’s microbiome. Similarly, Nile tilapia fed an experimental diet containing *L. lactis* PH3-05 (10^8^ CFU/g) showed the highest abundance of the Proteobacteria phylum [54]. It has also been documented that bacteria from the Bacteroidetes and Proteobacteria groups may originate from the aquatic environment [88]. The Bacteroidetes phylum of bacteria readily assimilates dietary carbohydrates, as members of this genus possess metabolic pathways to utilize them [89]. The genus *Sphingomonas* has been reported as an environmental microorganism, not as part of the gastrointestinal tract [90]. We consider that the presence of *Sphingomonas* in *A. tropicus* larvae results from the experiment’s surrounding environment and that it is displaced by bacteria belonging to the genera *Massillia* and *Sphaerotilus*, which showed the highest abundance and could be considered part of the indigenous microbiota during colonization. However, we do not yet know whether the gut microbial composition of *A. tropicus* larvae can be classified as autochthonous or allochthonous. Nevertheless, we can consider that the administration of *L. lactis* PH3-05 (10^8^ CFU/g) favored the abundance of proteobacteria in larvae fed with lower doses and the CD.

Our study provides new insights into the metabolic functions of gut microorganisms in *A. tropicus*. The identification of metabolic pathways related to carbohydrate (the lactic fermentation of hexoses and pentoses), waste (the Leloir pathway), and protein (proteolytic degradation and peptide transport) metabolism. Furthermore, the ability to synthesize lipids from fatty acids was discovered. Therefore, these findings contribute to a better understanding of the interaction between gut microorganisms and the physiology of tropical gar.

As can be seen, *L. lactis* PH3-05 can modify the microbiome and alter the abundance of bacteria from the phyla Bacterioides and Proteobacteria, particularly of the genera *Massillia*, *Sphaerotilus*, and *Sphingomonas*, enabling various pathways to manifest. These include (1) bacterial chemotaxis, allowing bacteria to move in response to nutrient gradients and other environmental stimuli [91]; (2) flagellar assembly, a transcriptional and post-transcriptional process that enables bacteria to move towards tissue colonization sites and perform multiple functions in host communication [92]; (3) fatty acid biosynthesis, revealing the diversity of the organization of the *pfa* genes, coding for a polyunsaturated fatty acid synthase complex [93]; (4) the biosynthesis of valine, leucine, and isoleucine, which are considered suitable targets for developing antibacterial agents [94]; and (5) the metabolism of dibasic acid C5-triphosphate, where a complex pathway has been described in different bacteria [95]. These findings reflected changes in the microbiome and enhanced *A. tropicus* PH3-05 larvae growth when fed with *L. lactis* (10^6−8^ CFU/g).

## 5. Conclusions

Feeding *A. tropicus* larvae with live *L. lactis* PH3-05, isolated as part of the native microbiome, significantly improves production values, digestive morphology, digestive enzyme activity, and immune system gene expression. Additionally, this probiotic modulates and strengthens various metabolic pathways of the microbiome, showing highly significant results when using doses of 10^6−8^ CFU/g in balanced feeds during weaning in the larval period. It is demonstrated that *L. lactis* PH3-05 can be considered a highly efficient probiotic that improves larval culture.

## Figures and Tables

**Table 1:** Composition of experimental diets with different concentrations of *Lactococcus lactis* and the control diet.

Ingredients (g kg diet^−1^)		*Lactococcus lactis* PH3-05 CFU/g		
	Control Diet	10^4^	10^6^	10^8^
Fish meal ^a^	350	350	350	350
Pork meal ^a^	270.9	270.9	270.9	270.9
Poultry meal ^a^	150	150	150	150
Starch ^b^	100	100	100	100
Fish oil ^a^	36.5	36.5	36.5	36.5
*Lactococcus lactis*	0	0.001	0.01	0.1
Wheat meal ^c^	37.6	36.6	37.59	37.6
Grenetin ^d^	20	20	20	20
Vit-min premix ^e^	15	15	15	15
Soy lecithin ^f^	15	15	15	15
Vitamin C ^g^	5	5	5	5
Chemical composition (g 100 g diet^−1^ Dry Matter)				
Crude protein	50.1	50.2	49.8	49.9
Crude lipid	14.2	14.1	13.9	14.2
Fiber	10.1	10.2	9.9	10.0
Ashes	14.0	13.9	14.1	14.2
Humidity	8.1	8.0	8.4	8.2
NFE	11.6	11.6	12.3	11.7

^a^ Marine and agricultural proteins S.A. de C.V., Guadalajara, Jalisco; ^b^ Pronat Ultra, Merida, Yucatan, Mexico; ^c^ GALMEX SA de CV, Villahermosa, Tabasco, México; ^d^ D’gari, food and diet products relámpago, S.A. de C.V. ^e^ Vitamin premix composition g/mg or International Units per kg of diet: Vitamin A, 10,000,000 IU; Vitamin D3, 2,000,000 IU; Vitamin E, 100,000 IU; Vitamin K3, 4.0 g; Thiamine B1, 8.0 g; Riboflavin B2, 8.7 g; Pyridoxine B6, 7.3 g; Vitamin B12, 20.0 mg; Niacin, 50.0 g; Pantothenic acid, 22.2 g; Inositol, 0.15 mg; Nicotinic Acid, 0.16 mg; Folic Acid, 4.0 g; Biotin, 500 mg; Vitamin C, 10.0 g; Choline 0.3 mg, Excipient q.s. 2 g; Manganese, 10 g; Magnesium, 4.5 g; Zinc, 1.6 g; Iron, 0.2 g; Copper, 0.2 g; Iodine, 0.5 g; Selenium, 40 mg; Cobalt 60 mg. Excipient q.s. 1.5 g; ^f^ Pronat Ultra, Yucatán, México; ^g^ ROVIMIX^®^ STAY-C^®^ 35–DSM, Guadalajara, México. NFE = Nitrogen-free extract: 100 − (% protein − % ether extract − % ash − %fiber).

**Table 2:** Primers used for qPCR analysis.

Target Gene	Gene Function	Primer Sequence (5′-3′)	Amplification Efficiency (%)	Amplicon Size (bp)	Reference
*muc-2*	mucus layer protein (mucin 2)	FW: GGCCTCCTCAAGAGCACGGTG RV: TCTGCACGCTGGAGCACTCAATG	90.94	100	[26]
*zo-2*	tight junction protein	FW: TACCCATGGAAAATGTGCCTCA RV: CGGGGTCTCTTCACGGTAA	95.29	88	[28]
*il-8*	pro-inflammatory cytokine	FW: ATATTCACTGGTGGGCGGAG RV: GTGCGGCCTGAGATTGTTT	94.18	369	[28]
*il-10*	anti-inflammatory cytokine	FW: TTATAAAGCCATGGGGGAGCTG RV: CTGCACAGTCTGCCTCTAGT	94.47	91	This study
*β-actin*	cytoskeletal actin	FW: GAGCTATGAGCTGCCTGAGTGG RV: GTGGTCTCATGAATGCCACAGG	97.10	119	[47]

**Table 3:** Indexes of the growth performance and survival rate of *A. tropicus* larvae fed diets supplemented with different concentrations of *L. lactis* PH3-05 (10^4^, 10^6,^ and 10^8^ CFU/g) compared with the control diet.

		*Lactococcus lactis* PH3-05 (CFU/g)		
	Control Diet	10^4^	10^6^	10^8^
Initial weight (g)	0.002 ± 0.007	0.002 ± 0.007	0.002 ± 0.007	0.002 ± 0.007
Final weight (g)	0.031 ± 0.002 ^b^	0.034 ± 0.0004 ^b^	0.040 ± 0.00 ^a^	0.041 ± 0.00 ^a^
Initial length (cm)	1.8 ± 0.18	1.8 ± 0.18	1.8 ± 0.18	1.8 ± 0.18
Final length (cm)	2.09 ± 0.05 ^b^	2.13 ± 0.12 ^a^	2.18 ± 0.09 ^a^	2.38 ± 0.09 ^a^
SGR (% d^−1^)	1.53 ± 0.07 ^b^	1.64 ± 0.07 ^b^	2.75 ± 0.15 ^a^	2.83 ± 0.003 ^a^
WG (%)	25.75 ± 1.35 ^b^	27.74 ± 1.46 ^b^	50.33 ± 3.50 ^a^	52.14 ± 0.06 ^a^
S (%)	31.11 ± 1.92 ^b^	33.75 ± 1.82 ^b^	46.36 ± 4.34 ^a^	32.56 ± 6.72 ^b^

SGR: specific growth rate; WG: weight gain; S: survival rate. Values are means ± SD. Significant differences are shown with different letters (*p* < 0.05).

**Table 4:** Digestive enzymatic activities of *A. tropicus* larvae fed diets supplemented with different concentrations of *L. lactis* PH3-05 (10^4^, 10^6,^ and 10^8^ CFU/g) compared with the control diet.

Activities (U mg protein^−1^)		*Lactococcus lactis* PH3-05 (CFU/g)		
	Control Diet	10^4^	10^6^	10^8^
Acid protease	490.79 ± 36.69 ^b^	490.27 ± 76.22 ^b^	716.99 ± 5.57 ^a^	235.30 ± 66.15 ^c^
Alkaline protease	41.14 ± 4.68 ^b^	44.45 ± 0.02 ^b^	55.45 ± 1.82 ^a^	37.46 ± 11.82 ^b^
Trypsin	0.12 ± 0.02 ^b^	0.22 ± 0.10 ^ab^	0.28 ± 0.05 ^ab^	0.35 ± 0.07 ^a^
Chymotrypsin	79.57 ± 2.70 ^ab^	87.45 ± 2.86 ^a^	89.06 ± 0.78 ^a^	65.86 ± 10.60 ^b^
Lipase	17.52 ± 0.21 ^b^	27.17 ± 1.21 ^b^	37.75 ± 0.3 ^a^	15.79 ± 8.64 ^b^
Leucine aminopeptidase	60. 21 ± 11.18 ^b^	83.66 ± 1.96 ^a^	81. 21 ± 3.58 ^ab^	71.72 ± 11.01 ^ab^

Values are means ± SD. Significant differences are shown with different letters (*p* < 0.05).

**Table 5:** Histological analysis of *A. tropicus* larvae fed with diets supplemented with different concentrations of *L. lactis PH3-* and 10^8^ CFU/g compared with the control diet.

Morphological Analysis		*Lactococcus lactis* PH3-05 (CFU/g)		
	Control Diet	10^4^	10^6^	10^8^
Area MMC (%/Area)	1.01 ± 0.43 ^b^	1.45 ± 0.14 ^ab^	1.91 ± 0.48 ^a^	1.94 ± 0.31 ^a^
Hepatocyte area (µm^2^)	5.48 ± 0.84 ^b^	5.72 ± 0.81 ^ab^	5.37 ± 0.66 ^b^	7.36 ± 0.25 ^a^
Enterocyte height (µm)	10.40 ± 0.40 ^d^	12.61 ± 0.22 ^c^	17.88 ± 0.40 ^a^	14.23 ± 0.33 ^b^

MMC: melanomacrophagic centers. Values are means ± SD. Significant differences are shown with different letters (*p* < 0.05).

## Data Availability

The data presented in this study are available upon request from the corresponding authors.

## Supplementary Materials

The following supporting information can be downloaded at: https://www.mdpi.com/article/10.3390/ani14182663/s1, Table S1: Antagonistic activity of *Lactococcus lactis* PH3-05 against pathogenic strains of fish.
