# Supplementary material for: The Indigenous Probiotic Lactococcus lactis PH3-05 Enhances the Growth, Digestive Physiology, and Gut Microbiota of the Tropical Gar (Atractosteus tropicus) Larvae
Source: Animals (Basel). 2024 Sep 13;14(18):2663. doi: 10.3390/ani14182663 (PMC11428600; doi:10.3390/ani14182663)
Supplement: Supplementary file 1 [file animals-14-02663-s001.zip › animals-3176499-supplementary.pdf]

Supplementary Table S1. Antagonistic activity of *Lactococcus lactis* against pathogenic fish strains.

Pathogenic bacteria	Zone of inhibition in diametre (mm)
	Lactococcus lactis
Aeromona hydrophila (NCIBM1134)	**1.3 ± 1.5**
Aeromona dhakensis (CAIM 1873)	**ND**
Aeromona ichtiosmia (CAIM 1876)	**ND**
Sthaphylococcus arlettae (CAIM 1658)	**13.3 ± 1.0**
Sthapylococcus arlettae (UJAT-02)	**12.8 ± 1.8**
Vibrio harveyi (CAIM 1622)	**ND**
Vibrio campbelli (GIBGEN 002)	**ND**
Photobacterium damselae (CAIM 192)	**ND**

Inhibition zone represented in mm; ND: No activity. Data are expressed as mean ± standard deviation (n=3).
